# Comprehensive Umbrella Review of the Management of Esophageal Anastomotic Leaks

**DOI:** 10.3390/jcm14092881

**Published:** 2025-04-22

**Authors:** Carlos M. Ardila, Daniel González-Arroyave, Jaime Ramírez-Arbeláez

**Affiliations:** 1 Department of Periodontics, Saveetha Institute of Medical and Technical Sciences, Saveetha Dental College and Hospitals, Saveetha University, Chennai 600077, India; 2 Biomedical Stomatology Research Group, Basic Sciences Department, Faculty of Dentistry Universidad de Antioquia U de A, Medellín 050010, Colombia; 3 Department of Surgery, Faculty of Medicine, Pontificia Universidad Bolivariana, Medellín 050031, Colombia; daniel.gonzaleza@upb.edu.co; 4 Department of Transplantation, Hospital San Vicente Fundación, Rionegro 054047, Colombia; jaime.ramirez@sanvicentefundacion.com

**Keywords:** esophageal anastomotic leaks, endoscopic vacuum therapy, self-expanding metal stents, systematic review, mortality reduction, treatment outcomes

## Abstract

**Background/Objectives**: Esophageal anastomotic leaks (EALs) are among the most feared complications following upper gastrointestinal surgery, particularly esophagectomy, given their profound impact on patient outcomes and healthcare resource utilization. This study aims to synthesize the evidence and determine the most effective interventions for achieving leak closure in patients with esophageal anastomotic leaks. **Methods**: This umbrella review followed PRISMA guidelines. A comprehensive search was conducted in PubMed, the Web of Science, Scopus, Google Scholar, Cochrane, and PROSPERO. Systematic reviews/meta-analyses on esophageal anastomotic leak management were included. The outcomes analyzed included leak closure success, mortality, complications, hospital stay, and costs. Data were synthesized narratively, with disagreements resolved by a third reviewer. **Results**: A systematic search identified 730 records, from which six systematic reviews and meta-analyses (evaluating 65 studies and 2186 patients) met the inclusion criteria. Most studies compared endoscopic vacuum therapy (EVT) and self-expanding metal stents (SEMSs) for EALs, with Germany contributing the majority. EVT consistently demonstrated superior leak closure rates and lower mortality compared to SEMSs, with pooled odds ratios favoring EVT. EVT also showed reduced complication rates, particularly fewer major adverse events, although with a slightly higher risk of post-therapy strictures. Hospital stay durations varied, with some studies reporting shorter treatment periods for EVT but no significant differences in the overall hospitalization length. Limited data suggested that EVT incurs higher treatment costs, largely due to intensive care unit stays. **Conclusions**: EVT is the most effective intervention for EALs, offering superior leak closure, lower mortality, and fewer complications. However, its economic impact requires further evaluation.

## 1. Introduction

Esophageal anastomotic leaks (EALs) are a severe complication following upper gastrointestinal surgery, particularly esophagectomy, with a significant impact on patient outcomes and healthcare resource utilization [1,2]. These leaks arise from dehiscence at the surgical anastomosis site, leading to the leakage of luminal contents into the mediastinum or surrounding tissues, often triggering severe inflammatory responses such as mediastinitis, sepsis, and multiorgan dysfunction [3]. The incidence of EALs remains high, ranging from 10% to 25% for cervical anastomoses and 3% to 25% for intrathoracic anastomoses, influenced by factors including patient-related variables (e.g., malnutrition, comorbidities, and immunosuppression), surgical technique (e.g., tension at the anastomosis or blood supply compromise), and postoperative management [3,4,5]. This high incidence and the associated morbidity underscore the urgent need for effective management strategies to address this challenging pathology [4,6].

Historically, managing EALs has been complex, with approaches such as surgical reintervention, conservative measures like antibiotics and drainage, and supportive care through parenteral or enteral nutrition. However, these methods often face limitations, including prolonged recovery and elevated mortality rates, particularly in complex cases [7,8]. The lack of a universally agreed-upon protocol further complicates treatment, often necessitating a multidisciplinary approach involving surgical, endoscopic, and medical teams. These challenges highlight the critical need for improved interventions to manage EALs effectively and reduce their devastating consequences [6].

Recent advancements in minimally invasive techniques have introduced endoscopic methods as promising tools for managing EALs, with self-expanding metal stents (SEMSs) and endoscopic vacuum therapy (EVT) gaining prominence [9,10]. SEMSs have been widely used, although they are associated with complications such as stent migration and pressure necrosis [9,10,11]. EVT, a newer approach, applies negative pressure to promote healing and has shown potential in reducing systemic inflammatory responses [9,10,12]. While both techniques offer advantages, their comparative effectiveness remains under investigation, with studies showing varied outcomes in different patient populations [6,13,14].

This umbrella review aims to synthesize evidence from systematic reviews and meta-analyses to compare the effectiveness of EVT, SEMSs, and surgical interventions for managing esophageal anastomotic leaks. Existing studies, such as those by Murray et al. [6] and Mandarino et al. [13], highlight the heterogeneity in findings regarding optimal treatment strategies, particularly in oncologic populations, while others like Jung et al. [14] and Monte Junior et al. [15] suggest that EVT may offer benefits in achieving leak closure [6,13,14,15]. By aggregating these data, this study seeks to identify the most effective strategies for leak closure while evaluating outcomes such as mortality, complications, hospital stay duration, and treatment costs. This work provides a comprehensive framework to address current evidence gaps and guide clinical decision making for EAL management.

Therefore, this study aims to synthesize the evidence and determine the most effective interventions for achieving leak closure in patients with esophageal anastomotic leaks while also assessing other important outcomes, including mortality, complications, hospital stay duration, and treatment costs.

## 2. Materials and Methods

### 2.1. Protocol and Registration

This umbrella review was conducted in accordance with the Preferred Reporting Items for Systematic Review and Meta-Analysis (PRISMA) guidelines [16]. A comprehensive search strategy was employed to identify systematic reviews and meta-analyses on the management of esophageal anastomotic leaks.

The search was conducted in multiple electronic databases, including PubMed, the Web of Science, Scopus, and Google Scholar, as well as systematic review databases such as the Cochrane Database of Systematic Reviews and PROSPERO. A comprehensive electronic database search was conducted, encompassing all records published from the inception of each database until February 2025, with no language restrictions applied. This umbrella review was registered in the PROSPERO database.

### 2.2. Search Strategy

A comprehensive search was conducted using the following terms: “oesophagectomy” AND “leak” OR “leakage” AND “management” OR “surgery” OR “esophagectomy” OR “esophagectomy” AND “leak” AND “manage” OR “Anastomotic Leak” AND “surgery” OR “therapy” AND “Upper Gastrointestinal Tract” AND “leak” AND “management” AND “endoscopic vacuum therapy” OR “endo sponge” OR “self-expanding metal stents” AND “Systematic review and Meta-analysis”.

As each database has its own unique search syntax and operators, the search strategies were adapted accordingly to ensure optimal retrieval of relevant articles.

### 2.3. Information Sources

Two independent reviewers performed the literature search, study selection, and data extraction. Disagreements were resolved through consensus or consultation with a third reviewer. Inter-rater reliability was assessed using the Kappa statistic, with scores above 0.90 indicating substantial agreement. The search strategy was documented and reported in accordance with the PRISMA statement [16].

### 2.4. Selection Criteria

The inclusion criteria consisted of systematic reviews and meta-analyses that investigated the management of esophageal anastomotic leaks, with a focus on the following:

Population: patients with esophageal anastomotic leaks.

Interventions: endoscopic vacuum therapy, self-expandable metal stents, and surgical revision.

Comparators: conservative management or any other intervention.

Outcomes: leak closure success, mortality, complications, hospital stay duration, and treatment costs.

Studies were excluded if they were any of the following:

Narrative reviews, editorials, case reports, case series, or letters to the editor.

Systematic reviews and meta-analyses that did not report on the leak closure success of the interventions.

Systematic reviews that lacked a clearly defined research question, a comprehensive search strategy, or a detailed description of the article selection process.

### 2.5. Data Extraction and Management

Two independent reviewers screened the titles and abstracts of all identified studies. Full-text articles of potentially eligible studies were then retrieved and independently assessed for inclusion by the same two reviewers. Disagreements regarding study inclusion were resolved through discussion with a third reviewer. Data extraction was performed independently by two reviewers using a standardized data extraction form.

### 2.6. Outcomes Measures

This umbrella review included systematic reviews and meta-analyses that assessed a range of clinically relevant outcomes. These outcomes included the following:

Leak closure success: the primary endpoint of the review, indicating the proportion of patients with successful healing of the anastomotic leak.

Mortality: all-cause mortality and mortality specifically related to the anastomotic leak or its management.

Complications: adverse events associated with the intervention or the underlying condition.

Hospital stay duration: the length of hospital stays for patients with anastomotic leaks, from admission to discharge.

Treatment costs: the overall economic burden of the intervention, including costs associated with hospitalization, procedures, and medications.

### 2.7. Risk of Bias Assessment

The quality of the included systematic reviews was assessed using AMSTAR 2 (A Measurement Tool to Assess Systematic Reviews 2) [17]. AMSTAR 2 includes 16 items, with seven critical domains (items 2, 4, 7, 9, 11, 13, and 15) essential for evaluating methodological rigor, such as protocol adherence, literature search strategy, risk of bias assessment, and publication bias investigation. The methodological quality was classified into four levels: high, moderate, low, and critically low. Two researchers independently conducted the assessment, and a third researcher resolved any disagreements to ensure consistency.

### 2.8. Data Synthesis and Analysis

Data from the included systematic reviews and meta-analyses were synthesized narratively.

### 2.9. Ethical Considerations

This umbrella review involved the secondary analysis of published data and did not involve direct interaction with human subjects. Therefore, ethical approval was not required.

## 3. Results

### 3.1. Systematic Review Selection

**Figure 1:**
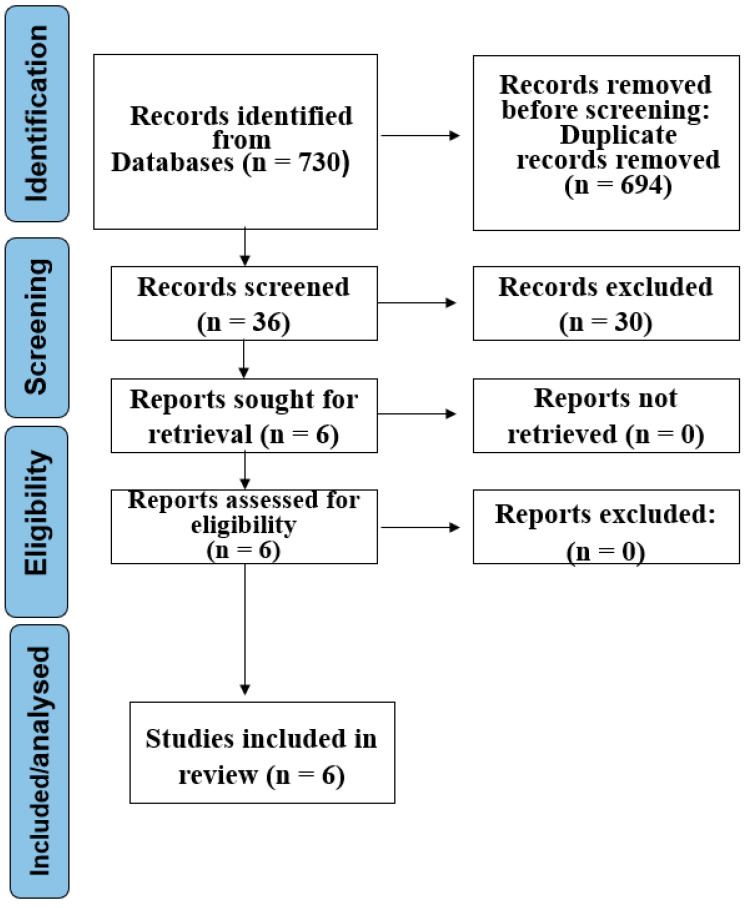
PRISMA flow diagram depicting the study selection process.

A comprehensive search yielded 730 initial records. Following the removal of duplicates and the application of predefined eligibility criteria, 36 studies were selected for full-text review. The exclusion criteria primarily included study designs other than systematic reviews and a lack of focus on interventions for achieving leak closure in esophageal anastomotic leaks. After a thorough review, six systematic reviews and meta-analyses met all inclusion criteria and were included in this umbrella review. Figure 1 presents the PRISMA flow diagram depicting the study selection process.

### 3.2. Overview of Systematic Review Findings

This umbrella review synthesized data from six systematic reviews and meta-analyses, collectively evaluating 65 studies and encompassing a total of 2186 patients. The included studies spanned diverse patient populations and interventions, with the majority focusing on the comparative effectiveness of EVT and SEMSs for managing esophageal anastomotic leaks, perforations, and fistulas. Germany emerged as the predominant country in these studies, featuring in 54 studies, with South Korea also represented in a few cases.

The distribution of patients across interventions varied across studies, as detailed in Table 1. Murray et al. [6] included the largest cohort (511 patients) with a broad range of interventions, including EVT, SEMSs, conservative management, and surgery. Other studies predominantly focused on the comparison of EVT and SEMSs.

The majority of the included studies investigated patients following oncological esophagectomy or gastrectomy. Cases of iatrogenic perforations and Boerhaave syndrome were also represented. Murray et al. [6] reported that stapled anastomoses were more prevalent than handsewn anastomoses (75.4% vs. 24.6%).

### 3.3. Primary Outcomes

#### Leak Closure Success

The primary endpoint of leak closure success was extensively analyzed across the included systematic reviews. Table 2 summarizes the key findings from these reviews, highlighting the comparative effectiveness of EVT and SEMSs. EVT consistently demonstrated higher closure success rates, as indicated by the pooled odds ratios (ORs) and relative risks (RRs) across studies. These findings strongly support EVT as the most effective primary intervention for esophageal anastomotic leaks, particularly in postoperative settings.

The effectiveness of EVT versus SEMSs did not vary significantly between different etiologies (e.g., perforations vs. leaks), as indicated in the subgroup analyses by Jung et al. [14].

### 3.4. Secondary Outcomes

#### 3.4.1. Mortality

Evidence consistently highlights the superiority of EVT over SEMSs and surgery in reducing mortality rates for esophageal anastomotic leaks. Systematic reviews and meta-analyses report significantly lower mortality risks with EVT, including a 12% reduction in all-cause mortality compared to SEMSs and pooled odds ratios consistently favoring EVT. While EVT demonstrated clear survival benefits, particularly for malignancy-associated leaks, some studies found no significant difference in in-hospital mortality between EVT and SEMSs. These findings establish EVT as a safer and more effective intervention, offering substantial survival advantages over alternative treatments (Table 3).

#### 3.4.2. Complications

As shown in Table 4, EVT consistently demonstrates a lower complication rate compared to other treatment modalities such as SEMSs, surgery, and conservative therapy. Monte Junior et al. [15] and Murray et al. [6] highlighted significant reductions in the RR and ORs for complications with EVT compared to SEMSs. Murray et al. [6] supported this finding, reporting an 18.2% complication rate for EVT, significantly lower than rates for SEMSs (39.8%), surgery (45%), and conservative therapy (28.6%). Similarly, Jung et al. [14] reported that adverse event rates associated with EVT were low. However, EVT was associated with a slightly higher rate of post-therapy strictures (14%, 95% CI: 10–20%), necessitating close follow-up. The systematic review by Scognamiglio et al. [18] noted that adverse events, including treatment-related complications and major complications, were numerically lower in the EVT group but did not reach statistical significance (OR 0.47, 95% CI:0.17 to 1.34 and OR 0.49, 95% CI: 0.17 to 1.40, respectively). However, EVT was associated with a statistically significant reduction in stricture formation after leak healing compared to SEMSs (OR 0.22, 95% CI: 0.08 to 0.62). The incidence of esophago-tracheal fistulas showed no significant differences between groups (OR 0.76, 95% CI: 0.12 to 4.68). Surgical review rates did not differ significantly between the EVT and SEMS groups (OR 0.44, 95% CI: 0.12 to 1.60) according to Scognamiglio et al. [18]. This suggests that the need for additional surgical interventions was similar across both treatment modalities. Rausa et al. [19] noted that complications were assessed in three studies, including 54 patients treated with EVT and 80 with SEMSs. Major complications occurred less frequently in the EVT group (5.6%) compared to the SEMS group (35%). In the EVT group, rare complications included bleeding from thoracic vessels (2.8%) and bronchoesophageal fistulas, both managed successfully with endoscopic or surgical interventions. In contrast, the SEMS group exhibited a higher incidence of major complications, with 28 patients affected. Surgical and endoscopic management was required in 14 cases, with minor complications managed conservatively in 10 patients.

Additionally, while EVT is associated with slightly higher rates of post-therapy stricture formation, its overall safety profile and reduced risk of complications make it a superior intervention for managing esophageal anastomotic leaks.

#### 3.4.3. Hospital Stay Duration

Hospitalization lengths varied significantly among interventions, as detailed in Table 5. Monte Junior et al. [15] and Mandarino et al. [13] found no significant differences in the duration of hospital stays between EVT and SEMSs. Mandarino et al. [13] observed that time in the intensive care unit (ICU) did not differ between the EVT and stent groups (OR: 1.32, 95% CI: −2.99–5.63). Jung et al. [14] and Scognamiglio et al. [18] highlighted that while EVT was associated with shorter treatment durations compared to SEMSs, no statistically significant differences in overall hospital stay were observed. However, Jung et al. [14] observed that EVT was associated with a shorter treatment duration, with a median reduction of approximately 12 days (95% CI: −18.59 to −5.21, *p* < 0.01) compared to SEMSs. Similarly, the review by Scognamiglio et al. [18] found that the duration of treatment was shorter with EVT than SEMSs, with a statistically significant pooled median difference of −11.57 days (95% CI: −17.45 to −5.69). Data on ICU stay durations were sparse, and no significant differences were observed (median difference of −0.5 days, 95% CI: −7.73 to 6.74) [18]. Murray et al. [6] noted EVT as having the longest mean hospital stay (51.9 days), exceeding SEMSs (46.6 days), surgery (38.9 days), and conservative therapy (35.9 days). These findings, while descriptive, were not statistically significant in the network meta-analysis. Compared to stenting, the meta-analysis did not reveal significant differences in hospitalization duration across intervention groups. Lastly, Rausa et al. [19] observed a pooled mean difference favoring shorter hospital stays with EVT, although this trend did not reach statistical significance.

#### 3.4.4. Treatment Costs

Treatment cost data for managing esophageal anastomotic leaks were inconsistently reported across the included reviews, limiting comprehensive economic comparisons between interventions. The systematic review by Scognamiglio et al. [18] included only one study that analyzed treatment costs [20], which reported that EVT incurred higher overall costs compared to SEMSs. In that study [20], the total cost for EVT was approximately 30% higher than for SEMSs, with around three-quarters of the expenses in both groups attributed to intensive care unit stays, driven by the longer hospitalization often required for EVT patients. Specifically, the study noted that ICU stays for EVT patients averaged 12 days compared to 8 days for SEMS patients, contributing to an estimated additional cost of USD 15,000 per patient for EVT due to extended ICU care [20]. Despite this, the direct costs of endoscopic management, including procedure-related expenses such as device costs and endoscopic sessions, were relatively low and comparable between the two approaches, with no significant difference observed (approximately USD 2500 for EVT versus USD 2300 for SEMSs per procedure) [20]. Additionally, EVT often requires more frequent endoscopic interventions—typically 5–7 sessions compared to 1–2 for SEMSs—which may further contribute to its higher overall cost despite similar per-procedure expenses. Indirect costs, such as those related to prolonged recovery and potential complications (e.g., secondary infections in EVT patients or stent migration in SEMS patients), were not adequately captured in the reviewed studies, highlighting a critical gap in understanding the full economic burden of these interventions. These findings underscore the need for more robust cost-effectiveness analyses to guide clinical decision making in EAL management.

#### 3.4.5. Consolidated Risks of Bias

As summarized in Table 6, the included systematic reviews exhibited varying levels of risk of bias. Methodological limitations were prevalent, primarily due to the reliance on retrospective studies and the absence of randomized controlled trials. Murray et al. [6] indirectly assessed the risk of bias, highlighting the potential impact of non-standardized measurements and variability in defect sizes. Mandarino et al. [13], using the Newcastle–Ottawa Scale [21], identified a moderate risk of bias in most studies, primarily attributed to the non-randomized design. Jung et al. [14] also employed the Newcastle–Ottawa Scale [21], finding that the majority of studies were of low or moderate quality, with limitations due to retrospective design and potential selection bias. Monte Junior et al. [15], utilizing the ROBINS-I tool [22], found a high risk of bias across all outcomes due to the lack of randomized trials and methodological limitations. Scognamiglio et al. [18] identified a low to moderate risk of bias in most studies, with one study exhibiting serious bias due to missing data. Rausa et al. [19], despite high-quality ratings based on the Newcastle–Ottawa Scale [21], acknowledged the inherent risk of selection bias due to the retrospective nature of the included studies. These factors should be carefully considered when interpreting the comparative effectiveness of EVT, SEMSs, and surgical interventions.

#### 3.4.6. Quality of Included Systematic Reviews

The methodological quality of the six studies included in the review was assessed using AMSTAR 2. Among these, four studies were categorized as high quality, while two were rated as critically low quality. A detailed summary of the AMSTAR 2 evaluation for these systematic reviews is provided in Table 7.

## 4. Discussion

This umbrella review synthesizes evidence from six systematic reviews and meta-analyses, encompassing 65 studies and 2186 patients, to provide a comprehensive evaluation of interventions for managing EALs, perforations, and fistulas. Our findings position EVT as the most effective intervention for achieving leak closure compared to SEMSs, surgery, and conservative management. EVT demonstrated consistent superiority across diverse etiologies, including postoperative leaks, iatrogenic perforations, and Boerhaave syndrome, highlighting its versatility as a primary treatment modality. This aligns with the broader clinical goal of improving patient outcomes in a condition known for its high morbidity and mortality, where effective leak closure is critical to preventing severe complications like mediastinitis and sepsis [6,13,14,15,18,19]. The ability of EVT to address a wide range of EAL etiologies underscores its potential as a first-line therapy, particularly in complex cases where other interventions may fall short.

Beyond leak closure, EVT also showed notable survival benefits, with reduced all-cause mortality compared to SEMSs and surgical interventions, particularly in malignancy-associated leaks. This survival advantage is likely due to EVT’s mechanism of action, which involves negative pressure to promote granulation tissue formation and reduce systemic inflammatory responses, thereby lowering the risk of fatal complications [14]. The reduced mortality rates observed with EVT are especially significant in the context of malignancy, where patients often have compromised health status and are more vulnerable to the consequences of treatment failure [13,15]. Additionally, EVT’s favorable safety profile, characterized by fewer complications compared to SEMSs, surgery, and conservative management, further supports its role as a preferred intervention. However, the occurrence of post-therapy strictures with EVT, while less frequent than with SEMSs, necessitates careful long-term follow-up to monitor for esophageal narrowing and ensure sustained patient recovery [6,13,14,15,18,19].

The primary outcome of leak closure success in this umbrella review aligns with findings from prior systematic reviews, which consistently report EVT’s superiority over SEMSs, surgery, and conservative therapy. Studies such as those by Monte Junior et al. [15], Murray et al. [6], Jung et al. [14], Mandarino et al. [13], Scognamiglio et al. [18], and Rausa et al. [19] all confirm that EVT achieves higher success in closing leaks, particularly in postoperative settings where the risk of dehiscence is elevated. This advantage is attributed to EVT’s ability to facilitate repeated endoscopic lavage and debridement, which helps manage local inflammation and promotes healing more effectively than SEMSs, which often require a longer interval to assess closure success [14]. However, some reviews highlight methodological challenges that temper these findings, such as non-uniform definitions of leak closure success across studies, which can introduce variability in reported outcomes [13,18]. Despite these discrepancies, the collective evidence strongly supports EVT as the most reliable intervention for achieving leak closure in EALs, offering a significant advancement over traditional approaches.

Regarding mortality, our analysis reinforces EVT’s survival benefits, with most studies reporting lower mortality rates compared to SEMSs and surgery. This finding is consistent across reviews, with Monte Junior et al. [15], Murray et al. [6], Jung et al. [14], and Rausa et al. [19] all noting EVT’s advantage in reducing all-cause mortality, particularly in high-risk patients with malignancy-associated leaks. Mandarino et al. [13] further emphasize EVT’s role in improving survival in such contexts, likely due to its ability to mitigate systemic complications through continuous monitoring and intervention. We propose that EVT’s survival advantage may stem from its mechanism of action, where negative pressure promotes granulation tissue formation and reduces systemic inflammatory responses, thereby lowering the risk of fatal complications like sepsis [14]. Additionally, EVT’s capacity for early intervention through scheduled endoscopic procedures can prevent prolonged ineffective treatment, which is particularly beneficial for vulnerable patients [15]. However, some studies, such as Scognamiglio et al. [18], found no clear difference in mortality between EVT and SEMSs, suggesting that factors like concomitant morbidities and quality of care may influence outcomes more than the intervention itself [18,19,20]. This discrepancy highlights the complexity of mortality as an outcome in EAL management, where patient-specific factors and institutional practices play a significant role in determining survival.

The safety profile of EVT is another key strength, with our review showing fewer complications compared to SEMSs, surgery, and conservative management. Monte Junior et al. [15], Murray et al. [6], Jung et al. [14], Mandarino et al. [13], and Rausa et al. [19] all report that EVT is associated with a lower incidence of adverse events, such as bleeding or perforation, which are more common with SEMSs due to its invasiveness and potential for device-related complications like dislocation. However, EVT is not without challenges: its requirement for frequent endoscopic interventions, while beneficial for monitoring and managing inflammation, can introduce periprocedural risks, such as those related to recurrent sedation [18,21,22,23]. Additionally, the slightly higher rate of post-therapy strictures with EVT compared to other interventions underscores the need for vigilant follow-up care to address potential long-term complications [14]. Despite these concerns, EVT’s overall safety profile makes it a well-tolerated option for most patients with EALs.

Hospitalization and treatment duration are critical considerations in EAL management, as they impact both patient recovery and healthcare resource utilization. Our review found that hospitalization durations for EVT and SEMSs were generally comparable, with no consistent advantage for either intervention across studies [6,13,14,15,18,19]. However, EVT demonstrated a notable benefit in shorter treatment duration, which may facilitate quicker recovery and reduce the overall burden on patients [14,18]. This advantage is likely due to EVT’s active management approach, which allows for faster healing through repeated interventions, as opposed to SEMSs, which often requires a more passive monitoring period [14]. Nevertheless, variability in patient populations and institutional protocols across studies complicates these findings, as some reviews report longer hospital stays with EVT due to its intensive care requirements [6]. This variability underscores the need for standardized care pathways to better evaluate the efficiency of EVT in clinical practice.

The economic implications of EVT versus SEMSs remain a complex issue, as cost data are inconsistently reported across systematic reviews. Our findings suggest that EVT incurs higher overall costs than SEMSs, primarily due to extended ICU stays and the need for more frequent endoscopic procedures [13,18,20,24,25,26,27]. Scognamiglio et al. [18] highlight that ICU stays are the primary cost driver for both interventions, with EVT’s longer hospitalization negating potential savings from its shorter treatment duration. Mandarino et al. [13] also note that EVT’s intensive follow-up requirements contribute to its higher costs, posing challenges for frail patients who may face additional risks from frequent procedures. However, Rausa et al. [19] suggest that EVT’s reduced complication rates and lower mortality could lead to long-term cost savings, despite its higher upfront expenses. These conflicting perspectives highlight the need for more comprehensive cost-effectiveness analyses to determine EVT’s financial feasibility across different healthcare systems.

The methodological limitations of this umbrella review and the included systematic reviews temper the interpretation of our findings. A common limitation across studies is the reliance on retrospective designs, which introduces potential bias and limits generalizability [6,13,14,15,18,19]. Murray et al. [6] and Mandarino et al. [13] note variability in defect sizes and non-standardized outcome measures as significant contributors to bias, while Jung et al. [14] and Monte Junior et al. [15] report moderate to high risks of bias due to the lack of randomized controlled trials (RCTs) [21,22]. Using AMSTAR 2, we rated four systematic reviews as high quality and two as critically low, reflecting variability in methodological rigor [18,19]. Scognamiglio et al. [18] further identify issues like missing data in some studies, which can skew results. These limitations highlight the challenges in synthesizing evidence for EAL management, where inconsistent definitions, heterogeneous techniques, and retrospective designs remain pervasive issues.

Despite these limitations, our umbrella review provides a comprehensive perspective on the comparative effectiveness of EVT, SEMSs, and surgery for EALs, consolidating findings from diverse studies to offer actionable insights for clinicians. The consistent superiority of EVT in achieving leak closure, reducing mortality, and minimizing complications positions it as a critical tool in improving patient outcomes, particularly in postoperative settings, where the risk of complications is high [6,13,14,15,18,19]. However, the methodological variability reported across reviews emphasizes the need for standardized protocols to ensure consistent application of EVT and accurate assessment of its outcomes [13,18]. The absence of prospective studies in many reviews further underscores the systemic reliance on lower-quality evidence, which limits the strength of current recommendations [6,15].

To address these gaps, future research should prioritize prospective, multicenter RCTs to validate EVT’s effectiveness and establish its role as a standard of care for EALs. Standardizing outcome measures, such as definitions of leak closure success and complication reporting, will be crucial for reducing heterogeneity and improving the reliability of future studies. Additionally, more robust economic evaluations are needed to assess EVT’s cost effectiveness, particularly in terms of optimizing ICU resource use and streamlining procedural efficiency [13,18,20,25,26,27]. Table 8 summarizes the key outcomes of this review, highlighting EVT’s clinical advantages alongside its economic challenges, and provides a framework for guiding future research and clinical decision making in EAL management.

## 5. Conclusions

This umbrella review provides a comprehensive synthesis of the comparative effectiveness of EVT and self-expanding metal stents (SEMSs) in managing esophageal anastomotic leaks. The findings consistently demonstrate that EVT is the superior intervention, achieving higher leak closure success rates and significantly reducing mortality and major complications compared to SEMSs. EVT also exhibited a favorable safety profile, with fewer overall complications despite a slightly increased risk of post-therapy strictures.

While hospitalization duration did not significantly differ between EVT and SEMSs, EVT was associated with shorter treatment durations. Cost-related data remain limited, but available evidence suggests EVT may impose a higher economic burden due to prolonged hospitalization. Nevertheless, its superior clinical outcomes justify its use as the primary therapeutic strategy for esophageal anastomotic leaks. These results support the growing preference for EVT in clinical practice and highlight the need for further cost-effectiveness studies to optimize treatment protocols.

## Figures and Tables

**Table 1:** Overview of findings from systematic reviews and meta-analyses.

Number of Studies	Patient Count	Interventions Studied/Patient Count	Key Findings	Reference
5	274	EVT n = 105 SEMS n = 169	EVT showed superior efficacy and safety compared to SEMSs.	[15]
12	511	EVT n = 123 SEMS n = 245 Conservative n = 87 Surgery n = 56	EVT is the recommended first-line treatment, with surgery considered for cases of large or complex leaks.	[6]
29	498	EVT n = 105 SEMS n = 169 Conservative n = 113 Surgery n = 111	EVT demonstrated a high success rate with lower rates of mortality, complications, and strictures compared to SEMSs.	[14]
8	357	EVT n = 152 SEMS n = 205	EVT revealed superior efficacy and safety compared to SEMSs in the overall population but similar efficacy in the oncologic subgroup.	[13]
7	383	EVT n = 148 SEMS = 190 Conservative n = 19 Surgery n = 26	EVT presented higher healing rates, shorter treatment duration, and lower stricture rates compared to stenting.	[18]
4	163	EVT n = 71 SEMS = 92	EVT demonstrated higher leak closure rates, shorter treatment duration, lower complication rates, and lower mortality compared to SEMSs.	[19]

EVT = endoscopic vacuum therapy; SEMS = self-expanding metal stent.

**Table 2:** Summary of leak closure success from systematic reviews and meta-analyses.

Outcome Measure (OR/RR)	Confidence Interval (95%)	*p*-Value	Key Findings	Reference
RR: 0.21	0.10–0.32	<0.001	EVT showed a 21% higher success rate in leak closure compared to SEMSs.	[15]
OR: 2.23	0.78–6.34	>0.05	This systematic review did not demonstrate significant differences between interventions; EVAC had the highest OR.	[6]
OR: 3.14	1.23–7.98	0.002	EVT showed a significantly higher closure success rate across 27 studies.	[14]
OR: 2.58	1.43–4.66	0.002	EVT demonstrated superior success rates, especially in leaks following esophagectomy.	[13]
OR: 2.47	1.30–4.73	<0.001	EVT was significantly more effective than SEMSs in promoting healing of esophageal leaks.	[18]
OR: 5.51	2.11–14.88	<0.001	EVT demonstrated a significant advantage over SEMSs in a pooled analysis of 134 patients.	[19]

RR = relative risk; OR = odds ratio; EVT = endoscopic vacuum therapy; SEMS = self-expanding metal stent.

**Table 3:** Summary of odds ratios and relative risks for mortality across systematic reviews and meta-analyses.

Effect Size	Confidence Interval (95%)	*p*-Value	Key Findings	Reference
EVT vs. SEMS RR: 0.12	0.21–0.03	0.006	EVT showed a 12% reduction in all-cause mortality.	[15]
EVT vs. SEMS OR: 0.43 Surgery vs. SEMS OR: 2.66	0.21–0.87 1.01–6.99	<0.05 <0.05	EVT significantly reduced mortality compared to SEMSs. Surgery was associated with a higher mortality risk.	[6]
EVT vs. SEMS OR: 0.39	0.18–0.83	0.01	EVT significantly reduced mortality compared to SEMSs.	[14]
EVT vs. SEMS OR: 0.47	0.24–0.92	0.002	EVT provided survival benefits, especially for malignancy-associated leaks.	[13]
EVT vs. SEMS OR: 0.58	0.26–1.30	>0.05	No significant difference was found in in-hospital mortality.	[18]
EVT vs. SEMS OR: 0.33	0.13–0.81	0.002	EVT significantly reduced in-hospital mortality compared to SEMSs.	[19]

RR = relative risk; OR = odds ratio; EVT = endoscopic vacuum therapy; SEMS = self-expanding metal stent.

**Table 4:** Summary of odds ratios and relative risks for complications in systematic reviews and meta-analyses.

Effect Size	Confidence Interval (95%)	*p*-Value	Most Frequent Complications	Reference
EVT vs. SEMS RR: 0.24	0.13–0.35	0.001	Migration, dislocation, stricture, fistula, and bleeding	[15]
EVT vs. SEMS OR: 0.27 Surgery vs. SEMS OR: 0.68 Conservative vs. SEMS OR: 0.40	0.14–0.54 0.15-0.35 0.14-0.14	<0.05 >0.05 >0.05	Migration, stricture, fistula, and bleeding	[6]
EVT vs. SEMS OR: 0.94	0.17–5.15	>0.05	Not reported	[14]
EVT vs. SEMS OR: 0.35	0.18–0.71	<0.05	Bleeding, perforation, ulcers, ingrowth, and esophageal–tracheal fistulas	[13]
EVT vs. SEMS OR: 0.47	0.17–1.34	>0.05	Stricture	[18]
EVT vs. SEMS OR: 0.38	0.11–1.27	0.011	Grade III–IV complications	[19]

RR = relative risk; OR = odds ratio; EVT = endoscopic vacuum therapy; SEMS = self-expanding metal stent.

**Table 5:** Hospital stay duration from systematic reviews and meta-analyses.

Outcome (Mean/Median Difference)	Confidence Interval (95%)	*p*-Value	Reference
EVT vs. SEMS 4.61 days	−3.59 to 12.80	>0.05	[15]
EVT vs. SEMS +4.71 Surgery vs. SEMS +7.46 Conservative vs. SEMS −0.0846	−4.48 to 13.7 −6.20 to 19.8 −12.6 to 12.1	>0.05 >0.05 >0.05	[6]
EVT vs. SEMS 2.81 days	−6.2 to 11.82	>0.05	[14]
EVT vs. SEMS 5.46 days	−3.87 to 14.79	>0.05	[13]
EVT vs. SEMS 6 days	−1.68 to 13.69	>0.05	[18]
EVT vs. SEMS +3.7 days	−6.6 to 14.1	>0.05	[19]

EVT = endoscopic vacuum therapy; SEMS = self-expanding metal stent.

**Table 6:** Summary of consolidated risks of bias across included studies.

Risk of Bias Assessment Tool	Study Quality	Key Sources of Bias	Key Findings	Reference
ROBINS-I	High risk across all outcomes	Lack of randomized clinical trials, poor standardization, and methodological rigor	High risk of bias due to methodological limitations across studies	[15]
Not specified	Not explicitly assessed	Variability in defect sizes and non- standardized measurements	Indirect assessment highlighted methodological limitations affecting bias	[6]
Newcastle–Ottawa Scale	15 studies: poor quality; 14: moderate quality	Retrospective design and lack of randomization caused selection bias	Overall findings limited by the predominance of low- quality, retrospective studies	[14]
Newcastle–Ottawa Scale	6 studies: fair/moderate risk; 2: poor quality	Non-randomized studies with moderate to high risk of bias	Fair-to-poor quality studies with moderate bias across non- randomized studies	[13]
ROBINS-I	6 studies: low to moderate risk; 1: serious risk	Missing data and retrospective study design	Mixed bias levels, with one study exhibiting serious bias due to missing data	[18]
Newcastle–Ottawa Scale	4 studies: high quality	Retrospective design and lack of randomization caused selection bias	Despite high quality ratings, inherent risk of selection bias was noted	[19]

**Table 7:** Methodological quality of the included systematic reviews based on AMSTAR 2.

Q1	Q2	Q3	Q4	Q5	Q6	Q7	Q8	Q9	Q10	Q11	Q12	Q13	Q14	Q15	Q16	Quality Rating	Reference
Yes	Yes	Yes	Yes	Yes	Yes	No	Yes	No	Yes	Yes	No	No	No	No	Yes	Critically low	[15]
Yes	Yes	Yes	Yes	Yes	Yes	Yes	Yes	Yes	Yes	Yes	Yes	Yes	Yes	Yes	Yes	High	[6]
Yes	Yes	Yes	Yes	Yes	Yes	Yes	Yes	Yes	Yes	Yes	Yes	Yes	Yes	Yes	Yes	High	[14]
Yes	Yes	Yes	Yes	Yes	Yes	Yes	Yes	Yes	Yes	Yes	Yes	Yes	Yes	Yes	Yes	High	[13]
Yes	Yes	Yes	Yes	Yes	Yes	Yes	Yes	Yes	Yes	Yes	Yes	Yes	Yes	Yes	Yes	High	[18]
Yes	Yes	Yes	Yes	Yes	Yes	No	No	Yes	No	Yes	No	No	Yes	No	No	Critically low	[19]

**Table 8:** Summary of key outcomes for esophageal anastomotic leak management interventions.

Outcome	Endoscopic Vacuum Therapy—EVT	Self-Expanding Metal Stents—SEMSs	Surgery
Leak closure	Most effective, superior across etiologies [6,13,14,15,18,19]	Less effective than EVT [6,13,14,15,18,19]	Less effective than EVT [6]
Mortality	Lowest rates, survival benefit [6,13,14,15,19]	Higher than EVT [6,13,14,15,19]	Highest among interventions [6]
Complications	Favorable safety profile, with fewer adverse events [6,13,14,15,18,19]	Higher rates, with more severe events [6,13,19]	Highest rates [6]
Hospital stay	Longer but shorter treatment duration [6,14,18]	Comparable to EVT [6,13,14,15,18]	Shorter than EVT [6]
Costs	Higher due to ICU stays, more procedures [13,18,20,25,26,27]	Lower than EVT [13,18,20,25,26,27]	Not consistently reported

## Data Availability

The datasets used and/or analyzed during the current study are available from the corresponding author upon reasonable request.
